# Longitudinal Associations Between Short-Term, Repeated, and Sustained Arts Engagement and Well-Being Outcomes in Older Adults

**DOI:** 10.1093/geronb/gbz085

**Published:** 2019-06-11

**Authors:** Urszula Tymoszuk, Rosie Perkins, Neta Spiro, Aaron Williamon, Daisy Fancourt

**Affiliations:** 1 Centre for Performance Science, Royal College of Music, London; 2 Faculty of Medicine, Imperial College London, UK; 3 Department of Behavioural Science and Health, University College London, UK

**Keywords:** Cohort study, Cultural participation, Eudaimonic well-being, Evaluative well-being, Hedonic well-being

## Abstract

**Objectives:**

This study investigated whether frequency of receptive arts engagement over 10 years contributes to experienced, evaluative, and eudaimonic well-being in older adults.

**Methods:**

We used repeated data of 3,188 respondents from Waves 2–7 (2004/2005–2014/2015) of the English Longitudinal Study of Ageing. We examined longitudinal associations between *short-term* (frequent engagement at one wave), *repeated* (frequent engagement at 2–3 waves), and *sustained* (frequent engagement at 4–6 waves) arts engagement and experienced, evaluative and eudaimonic well-being. We fitted linear and logistic regression models adjusted for baseline well-being and a number of sociodemographic, economic, health, and social engagement factors.

**Results:**

In the fully adjusted models, *short-term* engagement was not longitudinally associated with well-being, but *repeated* engagement with the theater/concerts/opera and museums/galleries/exhibitions was associated with enhanced eudaimonic well-being, and *sustained* engagement with these activities was associated with greater experienced, evaluative, and eudaimonic well-being.

**Discussion:**

Long-term frequent engagement with certain arts activities is associated with higher levels of happiness, life satisfaction, self-realization, and control/autonomy in older adults. These findings suggest that policies that facilitate older adults’ access to arts venues and activities, and support their continued engagement with them, may help to promote happy, fulfilling lives of an increasing segment of the population.

According to United Nations Department of Economic and Social Affairs, Population Division (2017) estimates, the global population aged 60 years and older is increasing faster than all younger age groups. Psychological well-being is prone to decline in old age due to commonly experienced events such as a partner’s death or disease, lone living, impoverished social interactions, low income, and progressive, age-related worsening health (NICE, 2016). There is thus a need to investigate sustainable ways of promoting well-being in later life.

Psychological well-being has been commonly recognized as a complex concept encompassing hedonic and eudaimonic elements (Ryan & Deci, 2001). Hedonic well-being comprises both experienced well-being (which entails affective aspects such as positive and negative affect, i.e., happiness or depressed mood), and evaluative well-being (which concerns perceptions of life quality, i.e., life satisfaction). Eudaimonic well-being includes multiple aspects related to optimal functioning, such as the basic psychological needs of relatedness, autonomy, and competence as well as engagement, purpose in life, and accomplishment (Ryan & Deci, 2001; Ryff, 2017). This multidimensional structure has been confirmed to capture elements important to the well-being of older adults (Vanhoutte, 2014), and reflects multiple priorities in healthy aging. Psychological well-being, in particular eudaimonic well-being, has also been associated with longer life expectancy and better health outcomes in old age (Cohen, Bavishi, & Rozanski, 2016; Ryff, 2017; Steptoe, Deaton, & Stone, 2015). Notably, the extent to which older adults perceive their lives as meaningful and purposeful has been robustly linked with extended longevity, improved physical and mental health, and social engagement (Cohen et al., 2016; Hill & Turiano, 2014; Steptoe & Fancourt, 2019).

There is now a growing body of evidence detailing the role of the arts and culture in promoting health and well-being throughout the life course (All-Party Parliamentary Group on Arts, Health and Wellbeing, 2017; Lomas, 2016; Royal Society for Public Health Working Group, 2013). Despite a lack of clear consensus on conceptualization and operationalization of arts engagement for population-based health research, arts engagement usually refers to broadly defined expressed or experienced human artistic creativity (All-Party Parliamentary Group on Arts, Health and Wellbeing, 2017; Davies et al., 2012). Across art forms (e.g., music, literature, the visual arts, and drama), increasing evidence suggests that engagement with arts-based activities can contribute to multiple aspects of experienced, evaluative, and eudaimonic well-being, such as enriched experience, enjoyment, meaning, bonding, and aesthetic appreciation (Lomas, 2016). Research in this field further commonly distinguishes between participatory engagement with the arts, which entails active creation of visual arts, drama, music or other art forms, and receptive engagement or participating as a spectator, such as attendance at arts-based events and venues. Both forms of engagement, often difficult to delineate clearly in real life, are believed to provide unique, individual encounters with the arts, which can inspire; aid exploration and expression of one’s identity, emotions, and talents; facilitate social interaction; and provide a temporary escape, rest, and catharsis (All-Party Parliamentary Group on Arts, Health and Wellbeing, 2017; Camic and Chatterjee, 2013).

Research on well-being benefits associated with arts engagement in older age, though, is currently dominated by short-term interventional studies involving participatory arts-based activities, and nonrandom samples of older adults. These studies find short-term well-being benefits for older adults who learn to make music (Perkins & Williamon, 2014), participate in community group singing (Coulton, Clift, Skingley, & Rodriguez, 2015), and museum-based programs (Thomson & Chatterjee, 2016; Thomson, Lockyer, Camic, & Chatterjee, 2018). Receptive engagement with the arts in adult populations has also been previously positively associated with life satisfaction (Cuypers et al., 2012; Wheatley & Bickerton, 2017), happiness (Wheatley & Bickerton, 2017), and quality of life (Nenonen, Kaikkonen, Murto, & Luoma, 2014) in cross-sectional studies using secondary large-scale data sources.

However, a number of key questions remain regarding the longevity and generalizability of the well-being benefits of arts engagement in old age. First, longitudinal explorations of the associations between arts engagement and well-being in representative samples are limited in number and report inconsistent findings (Menec, 2003; Węziak-Białowolska, 2016). Some studies have linked arts engagement with increases in happiness (Menec, 2003) and life satisfaction (Lakey, Smith, Oskala, & McManus, 2017), as well as lower odds of incident depressive symptoms over a decade (Fancourt & Tymoszuk, 2018), yet very few longitudinal studies have examined these associations specifically in older adults (Fancourt & Steptoe, 2018; Menec, 2003). Second, the majority of previous studies have been unable to examine distinct domains of well-being simultaneously (in particular eudaimonic well-being), thus further multidimensional analyses are needed. Third, some evidence suggests cultural stimulation is a “perishable commodity” (Johansson, Konlaan, & Bygren, 2001) in relation to some health outcomes, and can be subject to change similar to other lifestyle behaviors (O’Neill, 2010). Despite this, limited evidence exists on the frequency of arts engagement over time, and its association with long-term well-being.

To address these research gaps, we have used data from a large, nationally representative dataset of people aged 50 years and older (the English Longitudinal Study of Ageing [ELSA]) and investigated associations between *short-term*, *repeated*, and *sustained* engagement with receptive arts engagement (i.e., the frequency of visits to (a) the cinema; (b) art galleries, exhibitions, or museums; and (c) the theater, concerts, or the opera), and experienced evaluative and eudaimonic well-being outcomes over a 10-year period. The number of arts-based activities included in ELSA is relatively limited (when compared with, for instance, the lists generated for the Taking Part survey, conducted by the Department for Culture, Media, and Sport or by Davies et al., 2012). ELSA measures only engagement in receptive and usually ticketed arts activities occurring in state-supported or commercial cultural venues such as art galleries, cinemas, or concert halls, thus likely leading to underestimated and biased representation of arts engagement in the population (Davies et al., 2012; Taylor, 2016; Vanherwegen & Lievens, 2014). Nonetheless, it is the only nationally representative data set of older adults in England that permits the examination of at least some aspects of receptive arts engagement at regular intervals over time, and simultaneously contains data on multiple, different aspects of well-being (Vanhoutte, 2014).

## Methods

### Data and Study Sample

We used data from ELSA, a large, longitudinal cohort study representative of the English population of people aged greater than or equal to 50 years established in 2002 and designed as a stratified random sample of private households drawn from Health Survey for England. We specifically worked with data collected in Wave 2 (2004–2005) and biennially for the following decade (up to Wave 7; a total of 6 waves of data). Our sample consisted of participants with complete outcome data at Wave 7 as well as arts engagement information provided at a minimum of four of the waves between Waves 2 and 7 (*n* = 2,767). We used multiple imputation to account for missing data in covariates in Wave 2 (baseline), which yielded a total overall sample size of 3,188.

### Arts Engagement

Arts engagement was self-reported by the participants at Waves 2–7 and consisted of three items asking about the frequency of visits to (a) the cinema, (b) art galleries, exhibitions or museums, and (c) the theater, concerts, or the opera. Each arts engagement item was assessed on a 5-point scale: 0 (*never*), 1 (*less than once a year*), 2 (*once or twice a year*), 3 (*every few months*), and 4 (*once a month or more*). Due to a strong positive skew, this 5-point scale was subsequently coded as a binary variable of whether or not people engaged “frequently,” which we defined as engaging every few months or more. There was an approximate even split across the responses 0 (*engaging never or at most twice a year*) and 1 (*engaging every few months or more often*). Frequent arts engagement was then summed across six waves to generate the number of occasions at which frequent arts engagement was reported, with scores ranging from 0 to 6. The scores were further coded as 0 for *no or infrequent* arts engagement at all waves, 1 for *short-term* engagement (frequent engagement at one wave only), 2 for *repeated* engagement (frequent engagement at 2–3 waves), and *sustained* engagement (frequent engagement at 4–6 waves). For distribution of the engagement scores see Table 1.

**Table 1.** Descriptive Table of Participants Included in the Complete Cases Analysis, *n* = 2,767. Data Comes From ELSA Study Waves 2 (2004/2005) to 7 (2014/2005)

Covariates all measured at Wave 2	
Gender: female, *n* (%)	1,493 (54.0)
Age, mean (*SD*)	62.3 (7.1)
Ethnicity: Non-white, *n* (%)	26 (0.9)
In coupled relationship, *n* (%)	2,122 (76.7)
Education, *n* (%)	
Degree	525 (19.0)
A level/higher education	903 (32.6)
GCE and O level	629 (22.7)
No qualification	710 (26.7)
Employment status, *n* (%)	
Not in employment	1,474 (53.3)
Full time ≥35 hr/week	727 (26.3)
Part time	566 (20.5)
Eyesight problems: Yes, *n* (%)	207 (7.5)
Hearing problems: Yes, *n* (%)	443 (16.0)
Pain: Yes, *n* (%)	142 (5.1)
Long-standing illness, *n* (%)	824 (29.5)
No long-standing illness	1,380 (49.9)
Long-standing, not limiting illness	672 (24.3)
Long-standing and limiting illness	715 (25.8)
Social isolation score (0–9), mean (*SD*)	4.8 (1.8)
Participating in civic activities: Yes, *n* (%)	2,218 (80.2)
Frequency of arts engagement across the 10 years	
Engagement with the cinema, *n* (%)	
*No or infrequent* engagement at all waves	1,572 (56.8)
*Short-term* (frequent engagement at one wave)	316 (11.4)
*Repeated* (frequent engagement at 2–3 waves)	371 (13.4)
*Sustained* (frequent engagement at 4–6 waves)	508 (18.4)
Engagement with galleries/exhibitions/ museums, *n* (%)	
*No or infrequent* engagement at all waves	1,695 (61.3)
*Short-term* (frequent engagement at one waves)	317 (11.5)
*Repeated* (frequent engagement at 2–3 waves)	327 (11.8)
*Sustained* (frequent engagement at 4–6 waves)	428 (15.5)
Engagement with the theater/concerts/ opera, *n* (%)	
*No or infrequent* engagement at all waves	1,409 (50.9)
*Short-term* (frequent engagement at one wave)	486 (14.0)
*Repeated* (frequent engagement at 2–3 waves)	364 (13.1)
*Sustained* (frequent engagement at 4–6 waves)	608 (22.0)
Experienced well-being at Wave 7	
Positive affect: maximum vs lower score, *n* (%)	1,340 (48.4)
Evaluative well-being at Wave 7	
Life satisfaction, mean (*SD*)	26.6 (5.8)
Eudaimonic well-being at Wave 7	
Control/autonomy, mean (*SD*)	11.8 (2.6)
Self-realization, mean (*SD*)	10.8 (2.9)

SD = standard deviation.

### Well-Being Outcomes

#### Experienced well-being

Positive experienced well-being was measured with the 5-item Pleasure domain of the Control, Autonomy, Self-realisation, Pleasure scale (CASP-19) (Hyde, Wiggins, Higgs, & Blane, 2003). Each item is measured on a 4-point Likert scale (often = 3, sometimes = 2, not often = 1, never = 0), with higher scores indicating higher positive affective well-being. The scale displayed high internal validity in our sample (Cronbach’s α = .81). The scores were not normally distributed and were recoded as a binary variable: (1) maximum score of 15 versus (0) lower score. This yielded an approximately even split across the two responses (for the precise distribution of the scores see Table 1).

#### Evaluative well-being

Evaluative well-being was measured with the 5-item Diener’s life satisfaction scale (Diener, Emmons, Larsen, & Griffin, 1985), which consists of five items measuring overall satisfaction with life. Responses are summed to provide a score ranging from 5 to 35, with higher scores indicating greater life satisfaction. The scale displayed high internal validity in our sample (Cronbach’s α = .90).

#### Eudaimonic well-being

Eudaimonic well-being was measured with the 5-item Self-Realisation domain of the CASP-19 and the 5-item Control/Autonomy domain of a shortened version of the CASP-19 previously shown to have improved fit when analyzing the well-being of older adults (Vanhoutte, 2014). Each item is measured on a 4-point Likert scale (often = 3, sometimes = 2, not often = 1, never = 0), with higher scores indicating higher Self-Realisation or Control/Autonomy. Both scores ranged from 0 to 15 and displayed high-to-moderate internal validity in our sample (control/autonomy Cronbach’s α = .72 and self-realization Cronbach’s α = .83).

#### Covariates

Sociodemographic, economic, health, and social engagement covariates likely to confound the associations between exposure and outcome were measured at baseline (Wave 2). Sociodemographic covariates included age, gender, ethnicity (coded as white and non-white as ELSA is predominantly white British >98%) and being in a coupled relationship (in a couple vs without a partner). Socioeconomic position was assessed with (a) the highest educational attainment (categorized as university degree or equivalent, including NVQ4–NVQ5; A level/higher education or equivalent including NVQ3; The General Certificate of Education/O level qualification or equivalent including NVQ2; and other or no educational qualification), (b) employment status (full-time, part-time, not in employment), and (c) with net non-pension wealth quintiles. Net non-pension wealth measures accumulation of assets over the life span and is widely used as the most salient socioeconomic position indicator in the ELSA cohort. Participants’ health was measured with long-standing illness status (no long-standing illness, long-standing and non-limiting illness, long-standing and limiting illness). In addition, self-reported eyesight and hearing problems (coded as having problems if fair or poor eyesight or hearing reported) and experiences of moderate or severe pain, which could hinder one’s overall and arts engagement, were adjusted for. Participants registered as blind (*n* = 33) were dropped from the analyses due to possible different profiles of engagement in arts activities. Social engagement was measured with a composite score of frequency of contact (over the phone, E-mail, and face to face) with friends, children, and wider relatives (coded as +1 for each mode of contact and social tie, if contact occurred on a monthly basis or more frequently, with the score ranging 0–9), and a binary variable specifying engagement in any civic activities (including being a member of a political party or environmental group; a tenants or neighborhood watch group; a church or religious association; a charitable association; an education, arts, or music class; a social club, a sports, gym, or exercise class; or any other society).

### Statistical Analysis

We used logistic and linear regression models to assess the associations between different types of arts engagement measured over 10 years from Wave 2 to Wave 7 and well-being outcomes were measured in Wave 7. Model 1 adjusted for baseline (Wave 2) well-being, whereas Model 2 additionally adjusted for sociodemographic and socioeconomic covariates (gender, age, ethnicity, coupled relationship status, highest educational attainment, employment status, and net non-pension wealth), and Model 3 additionally adjusted for health variables (eyesight and hearing problems, long-standing illness status, and pain) and social engagement (social isolation index and civic activities). All well-being outcomes were examined in separate models. To correct for multiple comparisons and protect the results from Type I Error, we applied Bonferroni correction (0.05/12 = 0.004) and considered statistical significance to be most robust at the α value of less than 0.004.

We derived our analytical sample by first, restricting the data to participants with complete outcome data at Wave 7 (*n* = 3,695), and then to those with arts engagement information provided at a minimum of four of the waves between Waves 2 and 7 (*n* = 2,767). For those missing data in covariates at Wave 2 (baseline), we imputed the data using multiple imputation by chained equations (White, Royston, & Wood, 2011), with logistic, multinomial, ordinal, and linear regression according to variable type, generating 50 imputed data sets. The imputation model included all variables used in the analyses including the exposure, covariate, outcome variables, auxiliary variables assessing depressive symptoms and mobility problems, and the baseline survey weights. This yielded a total overall sample size of 3,188. The results of analyses did not vary between complete cases and imputed datasets. The findings presented here are based on the imputed dataset. All analyses were weighted to account for the stratified sampling design and to account of differential nonresponse.

We ran two sensitivity analyses. We excluded participants with (a) symptoms of depression at baseline (≥4 depressive symptoms scored on Center for Epidemiological Studies Depression 8-item version scale, *n* = 353) and (b) severe mobility issues at baseline (*n* = 465) as it was recognized that they might have different profiles of engagement. We found that the results did not vary substantially. These results can be found in Supplementary Tables 2 and 3. Characteristics of arts engagement profiles by sociodemographic, socioeconomic, health, and social engagement covariates are included in Supplementary Tables 4–6.

## Results

The sample characteristics are described in Table 1. Briefly, mean age at baseline was 62.3 years, 54.0% were women (Table 1). We found that 11.4%, 11.5%, and 14.0% reported *short-term* engagement with the cinema, galleries/exhibitions/museums, and the theater/concerts/opera, respectively. *Repeated* engagement with the cinema, galleries/exhibitions/museums, and the theater/concerts/opera was reported by 13.4%, 11.8%, and 13.1%. And 18.4%, 15.5%, and 22.0% of the participants reported *sustained* engagement with the cinema, galleries/exhibitions/museums, and theater/concerts/opera over 10 years of follow-up. We found demographic, socioeconomic, health, and social engagement differences in arts engagement profiles, with *sustained* engagement across all arts activities associated with younger age, higher educational attainment, full-time employment, being in the top wealth quintile, fewer health problems (eye or hearing problems, pain, long-standing illness), and higher social and civic engagement (Supplementary Tables 4–6).

The results from logistic and linear regression models presented in the main text (Table 2) include the full-adjusted models (Models 3). For other models (Models 1, 2, 3), see the Supplementary Table 1 in the Supplementary Material.

**Table 2.** Results From Logistic Regression Analyses Examining the Association Between Arts Engagement Activities and Odds of Maximum Experienced Well-Being Score and Linear Regression Models Examining the Remaining Well-Being Outcomes Using the Data Set With Imputed Missing Covariates (*n* = 3,188)

	Experienced well-being			Evaluative well-being			Eudaimonic well-being					
	Positive affect			Life satisfaction			Control autonomy			Self-realization		
	OR	95% CI	*p*	B	95% CI	*p*	B	95% CI	*p*	B	95% CI	*p*
Engagement with the cinema (reference: no or infrequent engagement at all waves)												
Short-term	0.80	0.61, 1.03	.08	0.001	−0.56, 0.56	.99	−0.18	−0.44, 0.08	.18	0.09	−0.20, 0.39	.54
Repeated	0.99	0.78, 1.26	.83	−0.01	−0.56, 0.54	.97	0.16	−0.10, 0.43	.23	0.09	−0.17, 0.36	.49
Sustained	1.14	0.91, 1.43	.25	−0.24	−0.74, 0.25	.34	0.06	−0.17, 0.29	.63	0.13	−0.10, 0.37	.28
Engagement with galleries/exhibitions/museums (reference: no or infrequent engagement at all waves)												
Short-term	0.98	0.76, 1.28	.93	0.32	−0.25, 0.90	.27	−0.001	−0.28, 0.27	.99	0.16	−0.12, 0.44	.26
Repeated	1.05	0.82, 1.35	.70	0.46	−0.08, 1.00	.10	**0.28**	**0.02, 0.54**	**.036**	**0.31**	**0.04, 0.58**	**.023**
Sustained	1.25	0.98, 1.61	.08	**0.76**	**0.28, 1.25**	**.002**	0.20	−0.04, 0.44	.10	**0.51**	**0.27, 0.76**	**<.001**
Engagement with the theater/concerts/opera (reference: no or infrequent engagement at all waves)												
Short-term	1.17	0.91, 1.51	.20	0.11	−0.45, 0.66	.71	0.08	−0.17, 0.33	.52	0.17	−0.10, 0.45	.21
Repeated	1.10	0.86, 1.41	.29	0.13	−0.42, 0.67	.66	**0.33**	**0.08, 0.58**	**.011**	0.27	−0.01, 0.54	.06
Sustained	**1.42**	**1.14, 1.77**	**.002**	0.43	0.02, 0.89	.06	**0.28**	**0.05, 0.51**	**.018**	**0.30**	**0.08, 0.53**	**.008**

*Notes*: All outcomes were entered and analyzed in individual models. Arts engagement was measured across ELSA Waves 2–7 (2004/2005–2014/2015); covariate data outcome data were measured at Waves 2 and 7, respectively. Participants who reported no arts engagement or engaging at most twice a year at all study waves; *short-term* (frequent engagement at one wave); *repeated* (frequent engagement at 2–3 waves); *sustained* (frequent engagement at 4–6 waves). All models are adjusted for Wave 2 well-being score, gender, age, ethnicity, coupled relationship status, highest educational attainment, employment status, and net non-pension wealth, eyesight and hearing problems, experiences of pain and chronic illness status, social isolation index, and civic activities. CI = confidence interval; OR = odds ratio. Bold values represent threshold value of statistical significance at 0.05.

### Short-Term Engagement (Frequent Engagement at One Wave)

#### Experienced, evaluative, and eudaimonic well-being

In the fully adjusted models, there was no difference in experienced, evaluative, and eudaimonic well-being outcomes between participants who reported *short-term* engagement in any of the arts activities and those who reported *no or infrequent* arts engagement at all waves (Table 2).

### Repeated Engagement (Frequent Engagement at 2–3 Waves)

#### Experienced and evaluative well-being

In the fully adjusted models, there was no difference in experienced and evaluative well-being outcomes between participants who reported *repeated* engagement in any of the arts activities and those who reported *no or infrequent* arts engagement at all waves (Table 2).

#### Eudaimonic well-being

In the fully adjusted models, *repeated* engagement with galleries/exhibitions/museums compared with *no or infrequent* engagement at all waves was associated with higher control/autonomy (B = 0.28, 95% confidence interval [CI]: 0.02, 0.54, *p* = .036) and self-realization (B = 0.31, 95% CI: 0.04, 0.58, *p* = .023). *Repeated* engagement with the theater/concerts/opera compared with *no or infrequent* engagement at all waves was associated with higher control/autonomy (B = 0.33, 95% CI: 0.08, 0.58, *p* = .011).

### Sustained Engagement (Frequent Engagement at 4–6 Waves)

#### Experienced well-being

In the fully adjusted model, *sustained* engagement with the theater/concerts/opera compared with *no or infrequent* engagement at all waves was associated with higher odds of maximum score of positive affect (odds ratio = 1.42, 95% CI: 1.14, 1.77, *p* = .002).

#### Evaluative well-being

In the fully adjusted models, *sustained* engagement with galleries/exhibitions/museums compared with *no or infrequent* engagement at all waves was associated with higher life satisfaction (B = 0.76, 95% CI: 0.28, 1.25, *p* = .002).

#### Eudaimonic well-being

In the fully adjusted models, *sustained* engagement with galleries/exhibitions/museums compared with *no or infrequent* engagement at all waves was associated with higher self-realization (B = 0.51, 95% CI: 0.27, 0.76, *p* < .001) in the fully adjusted model. S*ustained* engagement with the theater/concerts/opera compared with *no or infrequent* engagement at all waves was associated with higher control/autonomy (B = 0.28, 95% CI: 0.05, 0.51, *p* = .018) and self-realization (B = 0.30, 95% CI: 0.08, 0.53, *p* = .008).

### Sensitivity Analyses

Excluding participants with mobility issues (Supplementary Table 2) and depression at baseline (Supplementary Table 3) due to likely differences in engagement profiles in these groups did not materially change the results. The effect sizes for the associations between *repeated* and *sustained* engagement with galleries/exhibitions/museums seemed consistently larger in both sensitivity analyses. No consistent changes in effect sizes were found for *repeated* and *sustained* engagement with the theater/concerts/opera.

## Discussion

This study extends previously reported positive associations between arts engagement and subjective well-being for a population of older adults by examining *short-term*, *repeated*, and *sustained* arts engagement profiles over a 10-year time span and their longitudinal associations with distinct domains of experienced, evaluative, and eudaimonic well-being. The findings demonstrate that *sustained* engagement, taking place every few months or more over wthe majority of the 10-year follow-up period, is associated with long-term well-being benefits for older adults. Further, *repeated* engagement, taking place every few months or more across approximately 4–6 out of 10 years appears to be associated with eudaimonic well-being, although the magnitude of the association is weaker. We observe these associations only for engagement with certain arts activities, namely visits to galleries, exhibitions, museums, the theater, concerts, and the opera, but not the cinema. The findings, therefore, suggest that although receptive arts engagement is associated with well-being in older adults, the evidence for the association over time across the activities is the most robust when engagement is sustained.

This finding is in line with a previous study that linked both sustained cultural inactivity and decreasing cultural engagement to the same level of risk of impaired self-reported health (Johansson et al., 2001) and suggested that continued frequent engagement may be necessary for lasting health promotion. On the basis of these findings, others have proposed that arts engagement can be compared to adherence to lifestyle regimens such as physical activity habits that yield well-being benefits when sustained long-term but also when taken up after a period of inactivity (O’Neill, 2010). Although the importance of continued engagement with social and productive activities is recognized as being linked with better health (Johansson et al., 2001) and longevity (Thomas, 2012), only a few studies have investigated patterns of long-term leisure including arts activities engagement in aging populations (Agahi, Ahacic, & Parker, 2006; Strain, Grabusic, Searle, & Dunn, 2002), and none have linked them with subjective well-being. Nonetheless, evidence from previous small-scale intervention studies suggests that older adults consider regular participation in creative activities a good means of staying cognitively stimulated as well as seeing them as a “purposeful occupation,” and enriching daily routines (Swindells et al., 2013). Here, we further demonstrate that *sustained* engagement with *receptive* arts activities is particularly associated with positive experienced, evaluative, and eudaimonic well-being over time.

The focus on the multidimensional aspects of well-being was a particular feature of our research. In relation to experienced well-being, we found an association with *sustained* engagement with theater/concerts/opera. This is consistent with a previous prospective study linking baseline levels of engagement with music, art, and theater to enhanced happiness levels 6 years later (Menec, 2003) and in line with intervention studies reporting increases in older adults’ subjective experiences of pleasure, enjoyment, and other positive emotions as a result of music- and museum-based interventions (Clements-Cortés, 2017; Creech, Hallam, Varvarigou, McQueen, & Gaunt, 2013; Hays & Minichiello, 2005; Perkins & Williamon, 2014; Skingley, Martin, & Clift, 2016; Thomson & Chatterjee, 2016; Thomson et al., 2018). We further found that *sustained* engagement with galleries/exhibitions/museums was associated with greater evaluative well-being. This was in agreement with prospective (Lakey et al., 2017) and cross-sectional (Wheatley & Bickerton, 2017) analyses of UK-representative data sets. Our findings are further in line with a recent longitudinal study of the ELSA data set that found higher life satisfaction for participants who engaged with arts-based and education classes (Fancourt & Steptoe, 2018). However, they contrast those of longitudinal studies of Canadian and Swiss samples that reported null associations (Menec, 2003; Węziak-Białowolska, 2016). Direct comparisons with these studies are challenging due to differences in investigated populations and the life satisfaction measures used—the general life satisfaction single item measure (Węziak-Białowolska, 2016) and 20-item Life Satisfaction Index (Menec, 2003).

In relation to eudaimonic well-being, we found *sustained* and *repeated* arts engagement with galleries/exhibitions/museums and theater/concerts/opera was associated with increased control/autonomy and self-realization. These findings are in agreement with intervention studies that suggest that participating in creative and musical activities can foster feelings of control and autonomy as well as providing an opportunity for personal growth and engaging in challenging, simulating activities (Creech et al., 2013; Swindells et al., 2013). For instance, participatory musical activities have been previously described to help increase and maintain social, cognitive, and physical independence in older age (Hays & Minichiello, 2005), through, among other factors, the physical activity involved in participating in them (Creech et al., 2013). Furthermore, art and culture provide opportunities for self-directed engagement with activities one finds meaningful and absorbing, as well as sometimes cognitively and creatively challenging and, thus, can promote feelings of self-esteem, self-determination, competence, and accomplishment (Creech et al., 2013; Hays & Minichiello, 2005; Swindells et al., 2013). It is conceivable that attendance at arts events may contribute to eudaimonic well-being of older adults in a similar fashion.

Although our study clarifies specific associations between frequency and types of arts engagement and subjective well-being for a population of older adults, there are four areas in which further exploration is still warranted. First, this study focused on receptive arts engagement. Some evidence suggests potential differences in well-being and health outcomes between receptive and participatory arts engagement (Cuypers et al., 2012; Fancourt & Steptoe, 2018). However, these studies examined different aspects of engagement, including civic, religious, or community engagement, rather than strictly arts-based activity. Furthermore, some cross-sectional (Renton et al., 2012) and longitudinal (Węziak-Białowolska, 2016) studies examining both participatory and receptive arts-based engagement in relation to well-being and mental health outcomes in adult populations found no consistent differences in the associations. Further research is therefore needed to clarify how different participatory and receptive arts activities link to well-being outcomes in older adults. More generally, future research is needed to investigate mental health and health behavior profiles of older adults engaging with different arts pursuits, including those which are not captured in ELSA and which may be unticketed, informal, and representing a wider and more inclusive definition of arts engagement.

Second, in this study we found no associations with well-being outcomes for frequent engagement with cinema in the fully adjusted models. Engaging with cinema as an art form may have positive impact on subjective well-being and some evidence links cinema attendance with lower mortality rate (Konlaan, Bygren, & Johansson, 2000). However, previous studies also link screen time, such as watching television, with increased depressive symptoms, sedentary behavior, and other unfavorable health behaviors in older adults (Hamer, Poole, & Messerli-Bürgy, 2013). Investigation of different kinds of engagement with the arts through screens is necessary to disentangle the possible effects of related but perhaps experientially different pursuits.

Third, although our study found that long-term frequent engagement with certain arts activities is associated with higher levels of well-being, the majority of older adults in the ELSA sample were reporting no arts engagement. In addition, we identified demographic, socioeconomic, health, and social engagement differences in arts engagement profiles. Therefore, further research is needed to scrutinize nonparticipation, including investigation of structural and individual barriers. Linked with the previous points, such studies should also attempt to include a wider range of arts activities. Finally, with some preliminary evidence suggesting that arts-based activities and cultural spaces such as galleries and museums may help older adults to decrease feelings of loneliness, and to facilitate positive social interactions (Cohen et al., 2007; Creech et al., 2013; Hays & Minichiello, 2005; Todd, Camic, Lockyer, Thomson, & Chatterjee, 2017), future studies should examine these associations further, with a particular focus on loneliness and social isolation, which are known risk factors for low well-being in older adults (Shankar, Rafnsson, & Steptoe, 2015).

### Strengths and Limitations

This study answered calls for more prospective, population-based studies examining the associations between arts engagement and well-being. We used a sample from a large, nationally representative data set of older adults in England with arts engagement measured six times over 10 years, which enabled us to investigate *short-term*, *repeated*, and *sustained* arts engagement profiles of older adults. Despite the prospective design of this study, the causal direction of the associations cannot be inferred from our findings. As with any observational cohort study, biases stemming from nonrandom attrition and survival effects, as well as residual confounding, could explain observed estimates. Although we address some of these issues by adjustment for multiple covariates and potential differences in arts engagement profiles in sensitivity analyses, we found that participants who reported poorer health, lower well-being, and no arts engagement at baseline were more likely to drop out from Wave 7, and therefore do not appear in the analysis.

Our findings are particularly prone to residual confounding by socioeconomic differences in access and engagement with arts activities, which is further reinforced by the method of measurement of arts engagement in ELSA. Arts engagement questions in ELSA are skewed toward ticketed, and likely paid activities occurring in state-supported or commercial cultural venues, and fail to capture engagement with different art forms and in more informal settings—for example, at one’s at home, at community/cultural festivals, or digitally (Davies et al., 2012). This may have contributed to high rates (50%–60%) of reported no or infrequent engagement across the three types of receptive arts activities included in this study. Indeed, a previous study of the Taking Part data set confirms that over a half of the English population reports low levels of cultural participation, when it is measured through state-supported forms of culture (Taylor, 2016). The conceptualization of arts engagement in ELSA is not, however, atypical in its bias toward “highbrow interpretation” of what constitutes arts engagement (Lareau & Weininger, 2003; Vanherwegen & Lievens, 2014), its focus on receptive rather than participatory arts engagement (Vanherwegen & Lievens, 2014), or the omission of more popular forms of cultural participation (Taylor, 2016).

It is further important to acknowledge that habits of arts engagement in later life are likely to reflect one’s cultural capital “accrual” across the life course, starting in early life, and marked by intergenerational transmission and educational opportunities (Dimaggio & Mukhtar, 2004; Vanherwegen & Lievens, 2014). Sustained arts and social engagement in older age might further reflect cumulative health or socioeconomic advantage as well as other not easily modifiable antecedents (e.g., environmental) of greater access to arts activities. Indeed, we found that *sustained* (long-term, frequent) engagement across all arts activities was associated with higher educational attainment, better socioeconomic position, and fewer health problems. Nonetheless, engagement with the arts and culture can be a modifiable behavior as people’s practices, preferences, and proficiencies can change (Lareau & Weininger, 2003). There is increasing evidence to suggest that arts engagement can be a cost-effective, sustainable, and multimodal tool for health promotion when supported by adequate policies tackling access inequalities, and supporting older adults’ continued engagement (Camic & Chatterjee, 2013; Coulton et al., 2015).

Finally, the measure of well-being used in our study has limitations. CASP-19 was originally validated to measure quality of life in old age (Hyde et al., 2003), and captures only two elements (control and self-realization) relevant to eudaimonic well-being. Therefore, we were not able to investigate, among others, social elements of well-being despite evidence from previous studies suggesting salient interrelations between arts engagement, social connectedness, and well-being in older adults (Creech et al., 2013; Hays & Minichiello, 2005; Perkins & Williamon, 2014) or purpose in life despite well-established evidence on health and survival benefits associated with perceptions of purposeful life in older age (Hill & Turiano, 2014; Steptoe & Fancourt, 2019).

## Conclusion

Frequent visits to galleries, exhibitions, museums, the theater, concerts, or the opera sustained over the majority of a 10-year time span are associated with increases in experienced, evaluative, and eudaimonic well-being, specifically positive affect, life satisfaction, perceptions of control/autonomy over one’s life and self-realization. These findings, taken together with previous evidence of the importance of arts engagement for well-being in older adulthood, suggest that policies that facilitate access to certain arts venues and support older adult’s engagement with them, for example, through free admission schemes and investments in local cultural initiatives, may help to promote happy, fulfilling lives of an increasing segment of the population.

## Funding

The research reported in this article was supported by HEartS, a project funded by the UK’s Arts and Humanities Research Council to investigate the health, economic and social impact of the arts (grant ref. AH/P005888/1). U. Tymoszuk, R. Perkins, N. Spiro, and A. Williamon were funded through HEartS (AH/P005888/1), and D. Fancourt was supported by the Wellcome Trust (205407/Z/16/Z). The funding is provided by National Institute of Aging Grant R01AG017644 and a consortium of UK government departments coordinated by the Economic and Social Research Council.

## Author Contributions

Study design and critical input into manuscript: U. Tymoszuk, R. Perkins, N. Spiro, A. Williamon, and D. Fancourt. Data analyses: U. Tymoszuk and D. Fancourt. Manuscript preparation: U. Tymoszuk.

## Conflict of interest

None reported.

## Supplementary Material

gbz085_suppl_Supplementary_Tables
