# Supplementary material for: Longitudinal Associations Between Short-Term, Repeated, and Sustained Arts Engagement and Well-Being Outcomes in Older Adults
Source: J Gerontol B Psychol Sci Soc Sci. 2019 Jun 11;75(7):1609–19. doi: 10.1093/geronb/gbz085 (PMC7424284; doi:10.1093/geronb/gbz085)
Supplement: gbz085_suppl_Supplementary_Tables [file gbz085_suppl_supplementary_tables.docx]

Supplementary Tables

Table S1. Results from logistic regression analyses examining the association between arts engagement activities and odds of maximum experienced well-being score and linear regression models examining the remaining well-being outcomes using the dataset with imputed missing covariates, n=3,188. All outcomes were entered and analysed in individual models. Arts engagement was measured across ELSA waves 2 to 7 (2004/2005 – 2014/2015), covariate data outcome data were measured at waves 2 and 7 respectively.

|  | **Experienced well-being** | | | | **Evaluative well-being** | | | **Eudaimonic well-being** | | | | | |
| --- | --- | --- | --- | --- | --- | --- | --- | --- | --- | --- | --- | --- | --- |
|  | Positive affect | | | Life satisfaction | | | | Control-autonomy | | | Self-realisation | | |
|  | OR | 95% CI | p | B | | 95% CI | p | B | 95% CI | p | B | 95% CI | p |
| **Model 1: Engagement with the cinema** (reference: *no or* *infrequent engagement at all waves*) | | | | | | | | | | | | | |
| *Short-term* | 0.90 | 0.70, 1.16 | 0.42 | 0.14 | | -0.42, 0.69 | 0.63 | 0.01 | -0.26, 0.28 | 0.93 | 0.29 | -0.01, 0.58 | 0.06 |
| *Repeated* | 1.19 | 0.94, 1.50 | 0.14 | 0.23 | | -0.31, 0.78 | 0.40 | **0.48** | **0.21, 0.75** | **<0.001** | **0.38** | **0.11, 0.65** | **0.007** |
| *Sustained* | **1.48** | **1.19, 1.83** | **<0.001** | 0.19 | | -0.28, 0.65 | 0.43 | **0.56** | **0.34, 0.88** | **<0.001** | **0.61** | **0.38, 0.84** | **<0.001** |
| Model 2: Engagement with the cinema (reference: *no or* *infrequent engagement at all waves*) | | | | | | | | | | | | | |
| *Short-term* | 0.84 | 0.65, 1.08 | 0.18 | 0.11 | | -0.45, 0.68 | 0.69 | -0.12 | -0.39, 0.14 | 0.37 | 0.15 | -0.14, 0.44 | 0.32 |
| *Repeated* | 1.06 | 0.84, 1.35 | 0.61 | 0.11 | | -0.44, 0.66 | 0.69 | 0.25 | 0.02, 0.51 | 0.07 | 0.25 | -0.12, 0.42 | 0.28 |
| *Sustained* | 1.23 | 0.98, 1.54 | 0.08 | -0.13 | | -0.62, 0.36 | 0.61 | 0.13 | -0.09, 0.37 | 0.26 | 0.19 | -0.04, 0.43 | 0.11 |
| Model 3: Engagement with the cinema (reference: *no or* *infrequent engagement at all waves*) | | | | | | | | | | | | | |
| *Short-term* | 0.80 | 0.61, 1.03 | 0.08 | 0.001 | | -0.56, 0.56 | 0.99 | -0.18 | -0.44, 0.08 | 0.18 | 0.09 | -0.20, 0.39 | 0.54 |
| *Repeated* | 0.99 | 0.78, 1.26 | 0.83 | -0.01 | | -0.56, 0.54 | 0.97 | 0.16 | -0.10, 0.43 | 0.23 | 0.09 | -0.17, 0.36 | 0.49 |
| *Sustained* | 1.14 | 0.91, 1.43 | 0.25 | -0.24 | | -0.74, 0.25 | 0.34 | 0.06 | -0.17, 0.29 | 0.63 | 0.13 | -0.10, 0.37 | 0.28 |
| **Model 1: Engagement with galleries/exhibitions/museums** (reference: *no or* *infrequent engagement at all waves*) | | | | | | | | | | | | | |
| *Short-term* | 1.21 | 0.87, 1.45 | 0.38 | 0.44 | | -0.12, 1.00 | 0.13 | 0.18 | -0.10, 0.47 | 0.20 | 0.30 | **0.01, 0.60** | **0.043** |
| *Repeated* | 1.21 | 0.95, 1.55 | 0.12 | **0.60** | | **0.07, 1.14** | **0.027** | **0.48** | **0.22, 0.74** | **<0.001** | **0.43** | **0.16, 0.71** | **0.002** |
| *Sustained* | **1.57** | **1.25, 1.98** | **<0.001** | **1.00** | | **0.57, 1.44** | **<0.001** | **0.58** | **0.37, 0.81** | **<0.001** | **0.79** | **0.56, 1.03** | **<0.001** |
| Model 2: Engagement with galleries/exhibitions/museums (reference: *no or* *infrequent engagement at all waves*) | | | | | | | | | | | | | |
| *Short-term* | 1.02 | 0.80, 1.33 | 0.82 | 0.37 | | -0.20, 0.94 | 0.20 | 0.03 | -0.24, 0.31 | 0.82 | 0.18 | -0.10, 0.46 | 0.20 |
| *Repeated* | 1.11 | 0.86, 1.42 | 0.41 | 0.49 | | -0.05, 1.03 | 0.08 | **0.31** | **0.04, 0.57** | **0.023** | **0.34** | **0.07, 0.60** | **0.014** |
| *Sustained* | **1.34** | **1.04, 1.71** | **0.023** | **0.83** | | **0.35, 1.31** | **0.001** | **0.25** | **0.01, 0.49** | **0.041** | **0.55** | **0.31, 0.79** | **<0.001** |
| Model 3: Engagement with galleries/exhibitions/museums (reference: *no or* *infrequent engagement at all waves*) | | | | | | | | | | | | | |
| *Short-term* | 0.98 | 0.76, 1.28 | 0.93 | 0.32 | | -0.25, 0.90 | 0.27 | -0.001 | -0.28, 0.27 | 0.99 | 0.16 | -0.12, 0.44 | 0.26 |
| *Repeated* | 1.05 | 0.82, 1.35 | 0.70 | 0.46 | | -0.08, 1.00 | 0.10 | **0.28** | **0.02, 0.54** | **0.036** | **0.31** | **0.04, 0.58** | **0.023** |
| *Sustained* | 1.25 | 0.98, 1.61 | 0.08 | **0.76** | | **0.28, 1.25** | **0.002** | 0.20 | -0.04, 0.44 | 0.10 | **0.51** | **0.27, 0.76** | **<0.001** |
| **Model 1: Engagement with the theatre/concerts/opera** (reference: *no or* *infrequent engagement at all waves*) | | | | | | | | | | | | | |
| *Short-term* | **1.28** | **1.00, 1.64** | **0.048** | 0.05 | | -0.50, 0.60 | 0.86 | 0.18 | -0.08, 0.43 | 0.17 | 0.20 | -0.08, 0.48 | 0.16 |
| *Repeated* | **1.29** | **1.02, 1.65** | **0.036** | 0.25 | | -0.29, 0.79 | 0.36 | **0.59** | **0.34, 0.84** | **<0.001** | **0.45** | **0.17, 0.73** | **0.002** |
| *Sustained* | **1.77** | **1.45, 2.16** | **<0.001** | **0.67** | | **0.24, 1.09** | **0.002** | **0.63** | **0.41, 0.85** | **<0.001** | **0.56** | **033, 0.78** | **<0.001** |
| Model 2: Engagement with the theatre/concerts/opera (reference: *no or* *infrequent engagement at all waves*) | | | | | | | | | | | | | |
| *Short-term* | 1.22 | 0.95, 1.57 | 0.12 | 0.18 | | -0.38, 073 | 0.68 | 0.13 | -0.12, 0.38 | 0.31 | 0.21 | -0.06, 0.49 | 0.13 |
| *Repeated* | 1.18 | 0.92, 1.51 | 0.19 | 0.23 | | -0.31, 0.78 | 0.39 | 0.40 | 0.15, 0.65 | **0.002** | **0.32** | **0.04, 0.60** | **0.024** |
| *Sustained* | **1.56** | **1.25, 1.94** | **<0.001** | **0.59** | | **0.13, 1.04** | **0.011** | **0.38** | **0.15, 0.61** | **<0.001** | **0.38** | **0.16, 0.61** | **0.001** |
| Model 3: Engagement with the theatre/concerts/opera (reference: *no or* *infrequent engagement at all waves*) | | | | | | | | | | | | | |
| *Short-term* | 1.17 | 0.91, 1.51 | 0.20 | 0.11 | | -0.45, 0.66 | 0.71 | 0.08 | -0.17, 0.33 | 0.52 | 0.17 | -0.10, 0.45 | 0.21 |
| *Repeated* | 1.10 | 0.86, 1.41 | 0.29 | 0.13 | | -0.42, 0.67 | 0.66 | **0.33** | **0.08, 0.58** | **0.011** | **0.27** | **-0.01, 0.54** | **0.06** |
| *Sustained* | **1.42** | **1.14, 1.77** | **0.002** | 0.43 | | 0.02, 0.89 | 0.06 | **0.28** | **0.05, 0.51** | **0.018** | **0.30** | **0.08, 0.53** | **0.008** |
| **Reference:** Participants who reported no arts engagement or engaging at most twice a year at all study waves; ***short-term*** (frequent engagement at one wave); ***repeated*** (frequent engagement at 2-3 waves); ***sustained*** (frequent engagement at 4-6 waves)  **Model 1:** univariate + respective wave 2 well-being score; **Model 2**: Model 1 + gender, age, ethnicity, coupled relationship status, highest educational attainment, employment status, and net non-pension wealth; **Model 3**: Model 2+ health variables: eyesight and hearing problems, experiences of pain and chronic illness status and social engagement: social isolation index and civic activities | | | | | | | | | | | | | |

Table S2. Results from logistic regression analyses examining the association between arts engagement activities and odds of maximum experienced well-being score and linear regression models examining the remaining well-being outcomes using the dataset excluding n=465 participants with mobility issues, n=2,723. All outcomes were entered and analysed in individual models. Arts engagement was measured across ELSA waves 2 to 7 (2004/2005 – 2014/2015), covariate data outcome data were measured at waves 2 and 7 respectively.

|  | **Experienced well-being** | | | **Evaluative well-being** | | | **Eudaimonic well-being** | | | | | |
| --- | --- | --- | --- | --- | --- | --- | --- | --- | --- | --- | --- | --- |
|  | Positive affect | | | Life satisfaction | | | Control-autonomy | | | Self-realisation | | |
|  | OR | 95% CI | p | B | 95% CI | p | B | 95% CI | p | B | 95% CI | p |
| **Engagement with the cinema** (reference: *no or* *infrequent engagement at all waves*) ^a^ | | | | | | | | | | | | |
| *Short-term* | 0.77 | 0.58, 1.02 | 0.07 | 0.06 | -0.52, 0.63 | 0.84 | -0.17 | -0.44, 0.11 | 0.12 | 0.06 | -0.25, 0.36 | 0.72 |
| *Repeated* | 1.00 | 0.77, 1.30 | 0.99 | 0.06 | -0.50, 0.63 | 0.82 | 0.22 | -0.05, 0.49 | 0.11 | 0.11 | -0.17, 0.39 | 0.44 |
| *Sustained* | 1.14 | 0.89, 1.45 | 0.30 | -0.25 | -0.76, 0.27 | 0.35 | 0.06 | -0.17, 0.30 | 0.59 | 0.17 | -0.08, 0.41 | 0.19 |
| **Engagement with galleries/exhibitions/museums** (reference: *no or* *infrequent engagement at all waves*) ^a^ | | | | | | | | | | | | |
| *Short-term* | 0.98 | 0.74, 1.30 | 0.89 | 0.32 | -0.25, 0.89 | 0.28 | -0.04 | -0.28, 0.27 | 0.81 | 0.17 | -0.14, 0.48 | 0.27 |
| *Repeated* | 1.16 | 0.88, 1.51 | 0.30 | **0.59** | **0.01, 1.16** | **0.046** | **0.34** | **0.02, 0.54** | **0.016** | **0.40** | **0.12, 0.68** | **0.005** |
| *Sustained* | 1.30 | 0.99, 1.69 | 0.056 | **0.77** | **0.26, 1.29** | **0.003** | **0.27** | **-0.04, 0.44** | **0.035** | **0.53** | **0.27, 0.80** | **<0.001** |
| **Engagement with the theatre/concerts/opera** (reference: *no or* *infrequent engagement at all waves*) ^a^ | | | | | | | | | | | | |
| *Short-term* | 1.21 | 0.91, 1.51 | 0.18 | 0.12 | -0.46, 0.71 | 0.68 | 0.14 | -0.12, 0.40 | 0.29 | 0.25 | -0.03, 0.55 | 0.08 |
| *Repeated* | 1.09 | 0.86, 1.41 | 0.55 | -0.008 | -0.58, 0.56 | 0.98 | 0.25 | 0.01, 0.51 | 0.06 | 0.26 | -0.03, 0.56 | 0.08 |
| *Sustained* | **1.44** | **1.14, 1.77** | **0.002** | 0.47 | **-0.0001, 0.94** | **0.05** | **0.27** | **0.03, 0.51** | **0.028** | **0.32** | **0.09, 0.56** | **0.008** |
| **Reference:** Participants who reported no arts engagement or engaging at most twice a year at all study waves; ***short-term*** (frequent engagement at one wave); ***repeated*** (frequent engagement at 2-3 waves); ***sustained*** (frequent engagement at 4-6 waves)  ^a^ models adjusted for wave 2 well-being score, gender, age, ethnicity, coupled relationship status, highest educational attainment, employment status, and net non-pension wealth, eyesight and hearing problems, experiences of pain and chronic illness status, social isolation index and civic activities. | | | | | | | | | | | | |

Table S3. Results from logistic regression analyses examining the association between arts engagement activities and odds of maximum experienced well-being score and linear regression models examining the remaining well-being outcomes using the dataset excluding n=353 participants with depression at baseline, n=2,835. All outcomes were entered and analysed in individual models. Arts engagement was measured across ELSA waves 2 to 7 (2004/2005 – 2014/2015), covariate data outcome data were measured at waves 2 and 7 respectively.

|  | **Experienced well-being** | | | **Evaluative well-being** | | | **Eudaimonic well-being** | | | | | |
| --- | --- | --- | --- | --- | --- | --- | --- | --- | --- | --- | --- | --- |
|  | Positive affect | | | Life satisfaction | | | Control-autonomy | | | Self-realisation | | |
|  | OR | 95% CI | p | B | 95% CI | p | B | 95% CI | p | B | 95% CI | p |
| **Engagement with the cinema** (reference: *no or* *infrequent engagement at all waves*) ^a^ | | | | | | | | | | | | |
| *Short-term* | 0.75 | 0.58, 0.99 | 0.042 | 0.09 | -0.49, 0.68 | 0.75 | -0.12 | -0.39, 0.16 | 0.41 | 0.13 | -0.17, 0.43 | 0.39 |
| *Repeated* | 0.97 | 0.76, 1.25 | 0.83 | 0.08 | -0.49, 0.65 | 0.79 | 0.21 | -0.06, 0.48 | 0.13 | 0.14 | -0.13, 0.42 | 0.31 |
| *Sustained* | 1.22 | 0.96, 1.55 | 0.10 | -0.07 | -0.58, 0.45 | 0.80 | 0.10 | -0.13, 0.34 | 0.39 | 0.15 | -0.10, 0.39 | 0.24 |
| **Engagement with galleries/exhibitions/museums** (reference: *no or* *infrequent engagement at all waves*) ^a^ | | | | | | | | | | | | |
| *Short-term* | 1.01 | 0.77, 1.32 | 0.93 | 0.27 | -0.32, 0.87 | 0.37 | 0.008 | -0.29, 0.30 | 0.96 | 0.18 | -0.12, 0.48 | 0.24 |
| *Repeated* | 1.03 | 0.79, 1.34 | 0.83 | **0.59** | **0.03, 1.14** | **0.038** | **0.32** | **0.05, 0.58** | **0.020** | **0.42** | **0.15, 0.69** | **0.003** |
| *Sustained* | 1.23 | 0.95, 1.60 | 0.11 | **0.85** | **0.36, 1.35** | **0.001** | 0.14 | -0.10, 0.38 | 0.26 | **0.56** | **0.31, 0.82** | **<0.001** |
| **Engagement with the theatre/concerts/opera** (reference: *no or* *infrequent engagement at all waves*) ^a^ | | | | | | | | | | | | |
| *Short-term* | 1.16 | 0.89, 1.51 | 0.27 | 0.02 | -0.56, 0.62 | 0.94 | 0.10 | -0.17, 0.36 | 0.46 | 0.19 | -0.10, 0.48 | 0.21 |
| *Repeated* | 1.09 | 0.84, 1.41 | 0.55 | 0.10 | -0.46, 0.66 | 0.73 | **0.30** | **0.04, 0.56** | **0.025** | 0.23 | -0.06, 0.51 | 0.12 |
| *Sustained* | **1.46** | **1.16, 1.84** | **0.001** | 0.37 | -0.10, 0.85 | 0.12 | **0.27** | **0.04, 0.51** | **0.024** | **0.31** | **0.08, 0.54** | **0.009** |
| **Reference:** Participants who reported no arts engagement or engaging at most twice a year at all study waves; ***short-term*** (frequent engagement at one wave); ***repeated*** (frequent engagement at 2-3 waves); ***sustained*** (frequent engagement at 4-6 waves)  ^a^ models adjusted for wave 2 well-being score, gender, age, ethnicity, coupled relationship status, highest educational attainment, employment status, and net non-pension wealth, eyesight and hearing problems, experiences of pain and chronic illness status, social isolation index and civic activities. | | | | | | | | | | | | |

Table S4. Results from descriptive analyses examining arts engagement profiles by covariates, **n=2,767**. Engagement with the cinema was measured across ELSA waves 2 to 7 (2004/2005 – 2014/2015), covariates were measured at wave 2.

| **Engagement with the cinema** | | | | | |
| --- | --- | --- | --- | --- | --- |
| **Covariates all measured at wave 2** | No or infrequent | Short-term | Repeated | Sustained | *p* |
| **Gender**: female, n(%) | 795 (50.6%) | 182 (57.6%) | 223 (60.1%) | 293 (57.7%) | 0.001 |
| **Age**, mean (SD) | 63.17 (7.43) | 62.12 (6.79) | 61.40 (6.34) | 60.11 (5.88) | <0.001 |
| **In coupled relationship, n(%)** | 1,191 (75.8%) | 248 (78.5%) | 286 (77.1%) | 397 (78.2%) | 0.58 |
| **Education**, n(%) |  |  |  |  |  |
| Degree | 210 (13.4%) | 53 (16.8%) | 89 (24.0%) | 173 (34.1%) | <0.001 |
| A level/higher education | 495 (31.5%) | 111 (35.1%) | 126 (34.0%) | 171 (33.6%) |  |
| GCE and O level | 346 (22.0%) | 74 (23.4%) | 80 (21.5%) | 129 (25.4%) |  |
| No qualification | 521 (33.1%) | 78 (24.7%) | 76 (20.5%) | 35 (6.9%) |  |
| **Employment status**, n(%) |  |  |  |  |  |
| Not in employment | 933 (59.3%) | 153 (48.4%) | 176 (47.4%) | 212 (41.7%) | <0.001 |
| Full time ≥35 hours/week | 364 (23.2%) | 74 (23.4%) | 110 (29.7%) | 179 (35.3%) |  |
| Part time | 275 (17.5%) | 89 (28.2%) | 85 (22.9%) | 117 (23.0%) |  |
| **Wealth** |  |  |  |  |  |
| Top wealth quintile, n(%) | 372 (23.7%) | 90 (28.5%) | 124 (33.4%) | 213 (41.9%) | <0.001 |
| **Eyesight problems**: Yes, n(%) | 149 (9.5%) | 20 (6.3%) | 15 (4.0%) | 23 (4.5%) | <0.001 |
| **Hearing problems**: Yes, n(%) | 305 (19.4%) | 37 (11.7%) | 47 (12.7%) | 54 (10.6%) | <0.001 |
| **Pain**: Yes, n(%) | 105 (6.7%) | 13 (4.1%) | 10 (2.7%) | 14 (2.8%) | <0.001 |
| **Long-standing illness**, n(%) |  |  |  |  |  |
| No long-standing illness | 730 (46.5%) | 168 (53.2%) | 189 (50.9%) | 293 (57.7%) | <0.001 |
| Long standing, not limiting illness | 381 (24.2%) | 72 (22.8%) | 105 (28.3%) | 114 (22.4%) |  |
| Long standing and limiting illness | 461 (29.3%) | 76 (24.0%) | 55 (20.8%) | 101 (18.9%) |  |
| **Social isolation score (0-9)**, mean (SD) | 4.62 (1.82) | 5.03 (1.71) | 5.00 (1.91) | 5.23 (1.77) | <0.001 |
| **Participating in civic activities**: Yes, n(%) | 1,202 (76.5%) | 261 (82.6%) | 305 (82.2%) | 450 (88.6%) | <0.001 |

Table S5. Results from descriptive analyses examining arts engagement profiles by covariates, n=2,767. Engagement with galleries/exhibitions/museums was measured across ELSA waves 2 to 7 (2004/2005 – 2014/2015), covariates were measured at wave 2.

| **Engagement with galleries/exhibitions/museums** | | | | | |
| --- | --- | --- | --- | --- | --- |
| **Covariates all measured at wave 2** | No or infrequent | Short-term | Repeated | Sustained | *p* |
| **Gender**: female, n(%) | 919 (54.2%) | 170 (53.6%) | 167 (51.1%) | 428 (55.4%) | 0.68 |
| **Age**, mean (SD) | 62.65 (7.27) | 62.02 (6.84) | 61.76 (6.83) | 61.22 (6.33) | <0.001 |
| **In coupled relationship, n(%)** | 1,278 (75.4%) | 260 (82.0%) | 248 (75.8%) | 226 (78.5%) | 0.06 |
| **Education**, n(%) |  |  |  |  |  |
| Degree | 181 (10.7%) | 63 (19.9%) | 88 (26.9%) | 193 (45.1%) | <0.001 |
| A level/higher education | 546 (32.2%) | 115 (36.3%) | 106 (32.4%) | 136 (31.8%) |  |
| GCE and O level | 400 (23.6%) | 77 (24.3%) | 80 (24.5%) | 72 (16.8%) |  |
| No qualification | 568 (33.5%) | 62 (19.5%) | 53 (16.2%) | 27 (6.3%) |  |
| **Employment status**, n(%) |  |  |  |  |  |
| Not in employment | 949 (56.0%) | 161 (50.8%) | 169 (51.7%) | 195 (45.6%) | 0.006 |
| Full time ≥35 hours/week | 428 (25.2%) | 90 (28.4%) | 83 (25.4%) | 126 (29.4%) |  |
| Part time | 318 (18.8%) | 66 (20.8%) | 75 (22.9%) | 107 (25.0%) |  |
| **Wealth** |  |  |  |  |  |
| Top wealth quintile, n(%) | 363 (21.4%) | 103 (32.5%) | 112 (34.3%) | 221 (51.6%) | <0.001 |
| **Eyesight problems**: Yes, n(%) | 149 (8.8%) | 19 (6.0%) | 17 (5.2%) | 22 (5.1%) | 0.01 |
| **Hearing problems**: Yes, n(%) | 299 (17.6%) | 43 (13.6%) | 56 (17.1%) | 45 (10.5%) | 0.002 |
| **Pain**: Yes, n(%) | 103 (6.1%) | 15 (4.7%) | 12 (3.7%) | 12 (2.8%) | 0.02 |
| **Long-standing illness**, n(%) |  |  |  |  |  |
| No long-standing illness | 826 (48.7%) | 169 (53.3%) | 150 (45.9%) | 235 (54.9%) | 0.002 |
| Long standing, not limiting illness | 391 (23.1%) | 82 (25.9%) | 90 (27.5%) | 109 (25.5%) |  |
| Long standing and limiting illness | 478 (28.2%) | 66 (20.8%) | 87 (26.6%) | 84 (19.6%) |  |
| **Social isolation score (0-9)**, mean (SD) | 4.69 (1.82) | 4.91 (1.78) | 5.12 (1.83) | 5.09 (1.82) | <0.001 |
| **Participating in civic activities**: Yes, n(%) | 1,265 (74.6%) | 268 (84.5%) | 287 (87.8%) | 398 (93.0%) | <0.001 |

Table S6. Results from descriptive analyses examining arts engagement profiles by covariates, n=2,767. Engagement with the theatre/concert/opera was measured across ELSA waves 2 to 7 (2004/2005 – 2014/2015), covariates were measured at wave 2.

| **Engagement with the theatre/concerts/opera** | | | | | |
| --- | --- | --- | --- | --- | --- |
| **Covariates all measured at wave 2** | No or infrequent | Short-term | Repeated | Sustained | *p* |
| **Gender**: female, n(%) | 704 (49.9%) | 230 (59.6%) | 205 (56.3%) | 354 (58.2%) | <0.001 |
| **Age**, mean (SD) | 62.50 (7.30) | 62.96 (7.14) | 61.63 (6.63) | 61.61 (6.57) | 0.003 |
| **In coupled relationship, n(%)** | 1,055 (74.9%) | 307 (79.5%) | 283 (77.8%) | 477 (78.5%) | 0.13 |
| **Education**, n(%) |  |  |  |  |  |
| Degree | 151 (10.7%) | 64 (16.6%) | 81 (22.2%) | 229 (37.7%) | <0.001 |
| A level/higher education | 450 (31.9%) | 141 (36.5%) | 132 (36.3%) | 180 (29.6%) |  |
| GCE and O level | 301 (21.4%) | 96 (24.9%) | 98 (26.9%) | 134 (22.0%) |  |
| No qualification | 507 (36.0%) | 85 (22.0%) | 53 (14.6%) | 65 (10.7%) |  |
| **Employment status**, n(%) |  |  |  |  |  |
| Not in employment | 799 (56.7%) | 218 (56.5%) | 164 (45.0%) | 293 (48.2%) | <0.001 |
| Full time ≥35 hours/week | 364 (25.8%) | 82 (21.2%) | 108 (29.7%) | 173 (28.5%) |  |
| Part time | 246 (17.5%) | 86 (22.3%) | 92 (25.3%) | 142 (23.4%) |  |
| **Wealth** |  |  |  |  |  |
| Top wealth quintile, n(%) | 275 (19.5%) | 133 (34.5%) | 124 (34.1%) | 608 (43.91%) | <0.001 |
| **Eyesight problems**: Yes, n(%) | 139 (9.9%) | 26 (6.7%) | 17 (4.7%) | 25 (4.1%) | <0.001 |
| **Hearing problems**: Yes, n(%) | 266 (18.9%) | 68 (17.6%) | 46 (12.6%) | 63 (10.4%) | <0.001 |
| **Pain**: Yes, n(%) | 100 (7.1%) | 20 (5.2%) | 12 (3.3%) | 10 (1.6%) | <0.001 |
| **Long-standing illness**, n(%) |  |  |  |  |  |
| No long-standing illness | 636 (45.1%) | 195 (50.5%) | 201 (55.2%) | 348 (57.2%) | <0.001 |
| Long standing, not limiting illness | 339 (24.1%) | 92 (23.8%) | 93 (25.6%) | 148 (24.3%) |  |
| Long standing and limiting illness | 434 (30.8%) | 99 (255.7%) | 70 (19.2%) | 112 (18.4%) |  |
| **Social isolation score (0-9)**, mean (SD) | 4.59 (1.85) | 4.95 (1.69) | 4.96 (1.82) | 5.23 (1.77) | <0.001 |
| **Participating in civic activities**: Yes, n(%) | 1,023 (72.6%) | 321 (83.2%) | 317 (87.1%) | 557 (91.6%) | <0.001 |
